# Nutritional and fungal load dynamics of fresh brewers’ grain stored under aerobic conditions

**DOI:** 10.1186/s13568-022-01356-3

**Published:** 2022-02-01

**Authors:** Getu Kitaw, Mulisa Faji, Geberemariyam Terefe

**Affiliations:** Department of Animal Feeds and Nutrition, Holetta Agricultural Research Center, Holetta, Ethiopia

**Keywords:** Aerobic, Fungal, Storage durations, Temperature, Brewers’ grain

## Abstract

Brewers’ spent grain (BSG) is the amplest by-product of the brewing process. The fresh BSG is currently used as low-cost cattle feed due to its microbiological instability and high perishability. While recent research looked at the effects of storage time and temperature on the characteristics of wet brewers grains (WBG) as ruminant feeds. Three storage temperatures (15, 20, and 25 °C) and periods (2, 4 and 6 days) were arranged in a 3 × 3 factorial design. Surface spoilage was not apparent at 15 °C throughout the storage periods. Deterioration was not also observed at 20 °C until the fourth day of storage where slight mold growth was apparent. Extensive mold growth was detected late in the sixth day at 20 °C and continued manifestations up until the last day of storage at 25 °C. Changes in major nutrients, DM losses, and yeast and mold colony count were significantly affected by the interaction of storage temperatures and durations (P < 0.05). Except for samples stored at 15 °C, nutrients contents decreased concomitantly (exceptions are ADF, lignin, and loss in DM) with prolonged storage times (p < 0.05) and increasing temperatures (p < 0.05). Contrast analysis indicated that it would be safe to store under aerobic storage conditions and feed the WBG for dairy cattle.

## Key points

- The yeast and mold count increased with increasing storage times and temperatures
- It is safe to feed WBG to dairy an animal that is stored for less than 6 and 2 days at 15 and 20 °C, respectively.

## Introduction

Recycling and exploitation of brewing residues, such as brewers' spent grain (BSG), are critical goals for lowering energy consumption and residue disposal costs, as well as lowering the associated carbon footprint (Zupancic et al. 2017). The most common by-product of the brewing process is BSG (i.e., 85% of total by-products). From 100 kg of malt, 100 to 130 kg of fresh BSG (humidity, 70–80%) are obtained, equating to 21 to 22 kg BSG per hectoliter of beer brewed (Kunze 2004). According to Kaur and Saxena (2004), for every 100 kg of barley used in brewing, 170 kg of wet brewers' grains (WBG) are produced. BSG production has averaged 39 million tons per year around the world (Birsan et al. 2019).

The chemical compositions of BSG’s vary depending on barley cultivar, malting process, and brewing cereal quality and formulation (Gupta et al. 2010; Santos et al. 2003). BSGs, on the other hand, are high in dietary fiber, protein, and essential amino acids, as well as minerals, polyphenols, vitamins, and lipids (Fărcaş et al. 2014). WBG-fed dairy cows perform better than those fed dried brewers grains (Dhiman et al. 2003). Many dairy farms have chosen WBG as a cost-cutting measure. In a high humidity environment and at high temperatures, the storage time for WBG is typically short (Nofsinger et al. 1983). BSG is susceptible to microbial deterioration over a short period of time (i.e. 2 to 7 days) due to its high polysaccharide, protein, and moisture content (Wang et al. 2014; Gupta et al. 2010). Improper WBG storage causes a significant loss of DM and nutrients, as well as an unpleasant odor. It also encourages mold to produce mycotoxins, including aflatoxins (Asurmendi et al. 2013) and ochratoxin A (Amézqueta et al. 2009).

Currently, the most efficient way to exploit BSG is to sell it as livestock feed to local farmers. Breweries' long-term sustainability and environmental impact are jeopardized, however, because BSG production frequently exceeds demand for local feed (Mussatto et al. 2008). Temperature greatly varies throughout the dry and wet seasons poses as one of the most important environmental factors (Cohen 1985) that impact nutrient loss during storage. In the search for new ways to exploit BSG in foods and animal feed, as well as the pharmaceutical and cosmetic industries, research into new conservation systems, new uses, and enhancement technologies, as well as a better understanding of the use of microorganisms as biocatalysts, is critical. However, desired research findings do not exist and little is known about the nutrient changes in WBG stored at different temperatures and for different lengths of time. The objective of this experiment, therefore, was to evaluate the effects of storage duration and temperature on the chemical composition, in-vitro digestibility, and fungal load dynamics and recommend optimum storage duration under aerobic storage conditions for livestock producers located under varying geographical locations and climatic conditions in the country.

## Materials and methods

### Experimental locations, sampling procedures and measurements

The study was conducted on-station at Holetta Agricultural Research Center (HARC), Animal Nutrition and Dairy Microbiology Laboratories. The effect of aerobic storage conditions on brewer’s grain nutritional and fungal load dynamics was studied by considering three storage temperatures (15, 20 and 25 °C) and three storage duration (2, 4, and 6 days). Fresh brewery grain sample was obtained from a nearby Meta Abo brewery (40 km) for timely transportation of the samples to HARC Animal Nutrition Laboratory. Twenty-five kilograms of sample of WBG was collected using an ice box and stored in the lab using a deep freezer set at temperature of − 20 °C until the day the sample was ready for the next laboratory work. After thawing the frozen WBG, some 500 g of the sample was placed in pre-weighed sterile plastic beakers (500 ml capacity). All beakers were covered with 3 layers of cheesecloth to prevent rapid vaporization of moisture while retaining aerobic conditions in the beakers. Each WBG treatment was stored in a thermostatic incubator in five replications using the three storage temperatures and three storage times listed above in complete block design. A panel of three discussants assessed the physical features of samples that had been exposed to air during storage based on color, texture, odor, and the extent of mold cover. The panel's rating was converted to a numerical scale of 0 to 5, with 0 denoting no visible spoilage, l denoting minor mold growth, 2 denoting mold growth + discoloration, 3 denoting mold growth, discoloration, and surface collapse, 4 denoting mold growth, discoloration, and surface collapse + slight odor, and 5 denoting mold growth, discoloration, and surface collapse + offensive odor (Allen and Stevenson 1975). Initial and final weights of the beakers containing the samples were recorded to determine the DM loss taken at each incubation temperatures and storage periods. About 100 g of these samples on DM basis were subjected to freeze-drying for subsequent feed chemical composition (DM, ash, CP, NDF, ADF, permanganate lignin, DOMD) and growing molds and yeast colony count. The temperature regime and relative humidity condition (RH = 70%) used in this experiment were designed to roughly represent average daily temperatures and relative humidity conditions prevailing across the country where the WBG beneficiaries are located. The storage durations used in the present trial were also within the range of safe aerobic storage durations (2–7 day) recommended for fresh WBG under warm and cool tropical temperature conditions (Amaral-Philips and Hemken 2002; Thomas et al. 2010). The temperature and average relative humidity were controlled by allowing ± 1 °C and 1% fluctuation from the set temperature and relative humidity for all the incubations.

### Laboratory analysis

Brewers’ spent grain samples from different storage temperatures and duration were dried in a forced ventilation oven (55 °C for 72 h) and ground to pass through 1 and 2-mill Cyclotec sample mill screen (Tecator 1093, Tecator AB, Hoganas, Sweden). All samples were analyzed for DM, total ash, and crude protein (CP) using the procedure of AOAC (1990). Neutral detergent fiber (NDF), acid detergent fiber (ADF), and saturated potassium permanganate lignin were determined by the procedures of Van Soest and Robertson (1985). Tilley and Terry's (1963) two-stage *in-vitro* digestibility technique was employed to analyze and calculate the digestible organic matter in the dry matter of the samples. Metabolizable energy (ME) was estimated from the in-vitro organic matter in the dry matter digestibility (DOMD) as EME (MJ/kg) = 0.16 × DOMD (McDonald et al. 2002). Dry matter losses were calculated as a difference of DM for the fresh (control) WBG sample and the same samples that were subjected to aerobic and anaerobic storage treatments. Yeasts and molds were direct plates counted by pour plating of 25 g ground dried brewers’ grain samples dissolved in 225 ml of peptone water onto Potato Dextrose agar medium injected with 1 ppm per 100 ml of agar with chloramphenicol and streptomycin to restrict bacterial growth (FAO 1997). Plates were incubated aerobically at 28 ± 1 °C for 3 days and growing molds and yeast colonies were directly counted (MoH, 2010).

### Statistical analyses

The statistical model for lab experiment one was Y_ijk_ =  + C_i_ + L_j_ + CL_ij_ + e_ijk,;_ Where;Yijk = the response variable; = Overall mean; Ci = Effect of storage temperature; L_j_ = Effect of storage duration; CLij = Interaction effect; e_ijk_ = Random error. To compare the extent of feed quality deteriorations between the control/fresh and WBG samples that were conserved using aerobic and anaerobic storage techniques, orthogonal contrast analysis has been used. All data were subjected to analysis of variance using the general linear model (GLM) procedures of Statistical Analysis System, version 9.3 (SAS 2014). Mean separations were made using Least Significant Differences (LSD) analysis at P ≤ 0.05.

## Results

### Surface spoilage of fresh brewers’ grain stored under aerobic conditions

The extent of spoilage occurrence on the surface of the WBG stored aerobically under various storage temperature and duration conditions are presented in Table 1. When the WBG was stored at 15 °C no visible spoilage was observed up to the sixth day of storage. Deterioration was not also observed at 20 °C until the fourth day of storage where slight mold growth was apparent. Extensive mold growth was detected late in the sixth day at 20 °C and continued manifestations up until the last day of storage at 25 °C. Severe spoilage which was characterized by the worst spoilage rating of 5 was not observed at any given storage temperature and duration, although more deterioration was observed at 25 °C as storage duration increased.

**Table 1:** Surface spoilage ratings of fresh brewer’s grain stored at different storage temperatures and durations (relative humidity = 70%)

Storage temperatures (^o^ C)	Storage durations (days)		
	2	4	6
15	0	0	0
20	0	1	2
25	2	3	4

Ratings: *0* No visible spoilage, *l* Slight mold growth, *2* Mold growth + discoloration, *3* Mold growth + discoloration + surface collapse, *4* Mold growth + discoloration + surface collapse + slight odor, *5* Mold growth + deterioration + surface collapse + offensive odor

### Chemical composition and IVDOMD of brewer’s grain stored under aerobic conditions

Changes in the nutritional composition of all parameters measured and DM loss of WBG were significantly (P < 0.05) affected by the interaction of storage temperatures and durations (Table 2). At a temperature of 15 °C, storage duration did not have an effect on all parameters measured (P > 0.05). At temperatures of 20 and 25 °C, the DM, CP, NDF, in vitro digestibility decreased, while the ash, ADF, lignin and dry matter loss increased with increasing storage duration (P < 0.05). A similar trend of decreasing DM, CP, NDF, in vitro digestibility content, and increasing ash, ADF, lignin, and dry matter loss was observed with an increase in storage temperature.

**Table 2:** Effects of storage duration (days) and temperature (°C) on chemical composition, DOMD and dry matter loss of brewer’s grain (RH = 70%)

SD	ST	Chemical composition (g/kg for DM; g/kg DM for others and % for DML)							
		DM	Ash	CP	NDF	ADF	Lignin	IVDOMD	DML
2	15	242^a^	45^d^	264^a^	629^a^	253^ g^	67^e^	698^a^	0.1^d^
4	15	241^a^	46^d^	263^a^	629^a^	253^ g^	68^e^	697^a^	0.4^d^
6	15	240^a^	47^d^	261^a^	628^a^	254^ g^	68^e^	694^ab^	0.9^d^
2	20	240^a^	47^d^	261^a^	627^a^	270^f^	72^e^	687^b^	1.1^d^
4	20	218^b^	62^c^	232^b^	600^b^	314^d^	89^d^	630^d^	10.1^c^
6	20	201^c^	71^b^	212^c^	588^c^	333^c^	96^bc^	581^e^	17.2^b^
2	25	223^b^	61^c^	236^b^	576^d^	301^e^	91^ cd^	659^c^	8.0^c^
4	25	191^d^	75^b^	197^d^	567^ed^	346^b^	98^ab^	630^d^	21.3^a^
6	25	185^d^	83^a^	181^e^	558^e^	357^a^	102^a^	581^e^	23.7^a^
SEM		0.5	0.4	0.6	0.9	0.7	0.5	0.7	0.51

*SD* Storage date, *ST* Storage temperature, *DM* Dry matter, *CP* Crude protein, *NDF* Neutral detergent fiber, *ADF* Acid detergent fiber, *IVDOMD* in-vitro digestible organic matter in the dry matter, *DML* Dry matter loss, *SEM*  standard error of the mean

^a−g^Means within a column with different superscripts differ (P < 0.05)

The lower CP content was recorded from the BSG stored for 6 days at 25 °C temperature. While lower DM, NDF, IVDOMD and DM loss were observed for BSG stored for 6 days at 25 °C temperature. The higher lignin and ADF content were recorded for the BSG stored 6 days at 25 °C temperature.

Orthogonal contrast comparing the fresh WBG with those stored at combinations of three temperatures and storage durations is shown in Table 3. The chemical composition and digestibility values of WBG for the three storage periods at temperature 15 °C were similar to the fresh WBG (P > 0.05). Storage of WBG at 20 °C and for 2 days resulted in a significant reduction in digestibility and increase the ADF and lignin content (P < 0.05) as compared to the fresh WBG, while other values were similar between the two treatments. Storage at 20 °C and for the durations of 4 and 6 days and at 25 °C and all storage duration used in this study resulted in a significant effect on the values of all measured chemical composition and on in-vitro digestibility of OM, whereby the DM, CP, NDF and in vitro digestibility values were reduced while other values were increased after storage compared to the fresh WBG samples (P < 0.05).

**Table 3:** Contrast analysis for fresh brewer’s grain (T1) versus brewer’s grain stored at different storage temperatures (°C) and durations (days) (T2 to T10 (RH = 70%)

SD	ST	T	Contrast	Chemical composition (g/kg for DM and g/kg DM for others)						
				DM	Ash	CP	NDF	ADF	Lignin	DOMD
		T1		242	46	265	630	252	66	699
2	15	T2	T1–T2	0.2	0.4	1.0	0.5	− 0.9	− 0.8	1.2
4	15	T3	T1–T3	1.1	− 0.2	1.8	0.7	− 0.5	− 1.8	2.0
6	15	T4	T1–T4	2.1	− 0.8	3.3	1.2	− 1.7	− 2.2	4.8
2	20	T5	T1–T5	2.6	− 1.4	4.2	2.4	− 18.4*	− 5.7*	11.8*
4	20	T6	T1–T6	24.4*	− 16.8*	32.9*	29.9*	− 61.8*	− 22.7*	69.7*
6	20	T7	T1–T7	41.6*	− 24.8*	53.2*	41.2*	− 80.8*	− 29.5*	118*
2	25	T8	T1–T8	19.2*	− 14.8*	28.9*	53.9*	− 48.4*	− 24.7*	40.5*
4	25	T9	T1–T9	51.6*	− 28.8*	68.1*	62.1*	− 94.0*	− 31.8*	69.7*
6	25	T10	T1–T10	57.4*	− 37.0*	83.8*	71.3*	− 105*	− 36.1*	118*

*SD* Storage date (days), *ST* Storage temperature (°C), *T* Treatment, *DM* Dry matter, *CP*  Crude protein; *NDF*  Neutral detergent fiber, *ADF*  Acid detergent fiber, *DOMD* Digestible organic matter in the dry matter

Values for T1 are means and other values are mean differences between T1 and the respective treatments

*Contrast is significant (P < 0.05)

### Fungal load dynamics of brewer’s grain stored under aerobic conditions

Counts of yeast and mold for WBG samples stored at three temperatures and three durations were significantly affected (P < 0.05) by the interaction of storage temperature and storage duration (Table 4). At a storage temperature of 15 °C, the yeast and mold counts were similar (P > 0.05) for the three storage durations. At 20 °C, the mold count was not affected by storage duration (P > 0.05), while the yeast count was higher for 6 than 2 days of storage. Conversely, at 25 °C yeast count was similar for the three storage durations while the mold count was higher for 6 than rest of storage days. It generally appears that yeast and the mod count is lower for 15 °C than the other storage temperatures.

**Table 4:** Effects of storage duration (days) and temperature (°C) on yeast and mold counts of brewer’s grain (RH = 70%)

Storage durations	Storage temperatures	Fungal count (log10 CFU/g of WBG)	
		Yeast	Mold
2	15	4.8^e^	4.8^d^
4	15	5.5^de^	5.0^d^
6	15	5.7^cde^	5.3^ cd^
2	20	6.5^bcd^	5.6^bc^
4	20	7.2^ab^	5.7^bc^
6	20	7.7^a^	6.1^ab^
2	25	7.2^ab^	5.7^bc^
4	25	6.5^bcd^	5.7^bc^
6	25	6.8^abc^	6.6^a^
SEM		0.27	0.09

*WBG* Brewery spent grain, *cfu*  Coli form forming unit, *SEM* standard error of the mean

^a−g^Means with in a column with different superscripts differ (p < 0.05)

Orthogonal contrast of the fresh WBG with those stored at different combinations of temperatures and storage durations indicated that yeast count significantly increased at all combinations of temperature and storage durations except for WBG stored at 15 °C for 2 days (Table 5). On the other hand, mold count was not significantly affected for the three storage durations as 15 °C and for 2 days storage duration at 20 °C compared to the fresh WBG (P > 0.05), while the other temperature storage duration combinations significantly increased the mold count compared to the fresh WBG ( P < 0.05). Generally, there were no considerable changes (P > 0.05) in yeast and mold colony counts between the fresh and the stored WBG samples during the early hours of storage and low incubation temperatures.

**Table 5:** Contrast analysis of yeast and mold counts of fresh brewer’s grain (T1) versus brewer’s grain stored at different storage temperatures (°C) and durations (days) (T2 to T10 (RH = 70%)

SD	ST	T	Contrast	Fungal counts (log10 cfu/g WBG)	
				Yeast	Mold
		T1		4.24	4.08
2	15	T2	T1–T2	− 0.53	− 0.75
4	15	T3	T1–T3	− 1.23*	− 0.88
6	15	T4	T1–T4	− 1.48*	− 1.26
2	20	T5	T1–T5	− 2.22*	− 1.55
4	20	T6	T1–T6	− 2.95*	− 1.62*
6	20	T7	T1–T7	− 3.45*	− 1.99*
2	25	T8	T1–T8	− 2.98*	− 1.63*
4	25	T9	T1–T9	− 2.25*	− 1.60*
6	25	T10	T1–T10	− 2.54*	− 2.50*

*SD*  Storage date (days), *ST*  Storage temperature (°C), *T*  Treatment, *WBG*  Brewery spent grain, *cfu*  Coliform forming unit

Values for T1 are means and other values are mean differences between T1 and the respective treatments

*Contrast is significant (P < 0.05)

## Discussion

A higher level of air exposure (longer storage duration) and higher storage temperature in the present study were the two most important features that characteristically contributed to aerobic changes of WBG under storage. McDonald et al. (1991) noted that deteriorations in air-exposed silage were majorly manifested by an elevation in temperature, a change in the odor, and the appearance of mold. The WBG stored under aerobic conditions for two days at temperatures below 20 °C showed visible changes, including an unpleasant odor, surface cracking, and color changes, which could have been caused by the rapid proliferation of yeast and molds, resulting in large nutrient losses in the fresh brewery grain sample used in this study. Feeding such spoiled material (> 5.00 log CFU/g DM of yeast and mold, which according to GMP 2008) might be a risk to the health of the animals and humans through carryover effects due to the likely production of mycotoxins such as aflatoxins (Asurmendi et al. 2013; Souza et al. 2012).

The aerobic deterioration of WBG stored at high temperature and longer durations observed in the present study could be major factors responsible for high losses in nutritional components and reduction in DM digestibility. Hao et al. (2015) reported reduced IVDMD and DM loss with the extended exposure time, which is consistent with the current finding. In a related study conducted with distillers’ grain stored in air-exposed bunker silos (Baskett et al. 2009) storage DM loss of 9.6% was reported. Marston et al. (2009) also observed a sharp drop in DM by 37.7% for uncovered, aerobically stored fresh WBG, with the larger DM loss appeared to have been associated with the longer storage periods. The incremental changes in ash with storage period and temperature over the control WBG have also been noted earlier by Marston et al. (2009), which could be associated with the loss in organic matter that proportionally increased the ash content.

As opposed to an earlier finding by Wang et al. (2014), a markedly higher reduction in CP value relative to the control WBG was observed in this study, which could be attributed to ammonia losses from proteolysis by increased mold and yeast populations during aerobic fermentation and subsequent ventilation (Zopolatto et al. 2009). ADF was generally increasing consistently while NDF was on the opposite trend as storage periods and temperatures were advancing beyond 15 °C, indicating that the DM loss from the current trial was partly derived from the fresh WBG hemicellulose contents. This finding agrees with the report by Marston et al. (2009) but was in contrast with the findings of Turner et al. (2002), who observed that all of the cell wall components constantly increased during the entire storage period and temperature conditions. The variation could be explained by the existence of more easily degradable hemicellulose in WBG in the current study than in the forages that the researchers used. The increased ADF and lignin contents during aerobic storage can be justified by the relative decrease in the other cell contents (Santos et al. 2010).

The growth of microorganisms was unaffected by storage duration at a storage temperature of 15 °C. At higher temperatures of 20 °C and 25 °C, the effect was seen at 2 days of storage. In the studies of Wang et al. (2014) and Coskuntuna et al. (2010), WBG samples that were exposed to 20 to 30 °C temperature deteriorated more than those exposed to > 30 °C. According to Higgins and Brinkhaus (1999), this phenomenon is most likely caused by the sigmoid growth nature of both microorganisms and their mycotoxins production in stored WBG. Mycotoxins and secondary metabolites produced by fungi are extremely harmful to both human and animal health (CAST 2003). The extensive aerobic deterioration that led to the higher DM/OM losses with higher storage temperatures and storage durations in the present study might have been triggered by the large numbers of yeasts and molds proliferated as a result of residual water-soluble carbohydrates in the fresh WBG (Wang et al. (2014). Furthermore, the early plateau observed at 20 °C in the current trial could be due to the WBG samples' inherently high moisture content (Ashbell et al. 2002). Except for control and WBG samples stored in the early phases (2 days) for yeast and (6 days) for mold at a lower temperature (15 °C), both yeast and mold colony counts for the remaining WBG samples exceed the limit > 5.00 log CFU/g DM, which according to GMP, (2008) and Dairy One (2017) is a sign for aerobic instability in stored feeds.

There are limited studies of the effect of temperature and storage duration on the feed nutritional quality of WBG. On the basis of major feed and microbial quality data generated from the current study, it would be possible to advise dairy producers and recommend optimum storage durations under aerobic conditions for fresh WBG stored under varying environmental storage temperature conditions. Accordingly, taking the control as a benchmark, it would be safe to store under aerobic storage conditions and feed the WBG for dairy cattle provided that it is stored for less than 6 and 2 days at 15 and 20 °C, respectively.

In an attempt to determine optimum storage durations for fresh WBG samples stored under varying aerobic storage duration and temperature conditions, it would be safe to store and feed fresh WBG under local conditions provided that it is stored for less than 6 and 2 days at 15 °C and 20 °C, respectively (P < 0.05) without being exposed to aerobic deteriorations. To ensure efficient utilization of available WBG for dairy cattle under local conditions, further research engagements are required in the years to come. Accordingly, future research planning with WBG shall consider additional storage durations and temperatures, humidity conditions and biochemical test in a way that it precludes mycosis and further deteriorations in feed quality of WBG stored under aerobic storage conditions.

## Data Availability

The datasets generated during and analyzed during the current study are available from the corresponding author on reasonable request.
